# A Lightweight Keyword Spotting Method Using a Convolutional Spiking Neural Network with Learnable Synaptic Delays

**DOI:** 10.3390/s26144514

**Published:** 2026-07-16

**Authors:** Xiaohuan Li, Yi Liu, Libo Zheng

**Affiliations:** College of Integrated Circuit Science and Engineering, Nanjing University of Posts and Telecommunication, Nanjing 210023, China; 2022221002@njupt.edu.cn (X.L.); zhenglibonjupt@outlook.com (L.Z.)

**Keywords:** keyword spotting, CSNN, streamable convolutional encoding, synaptic delays, lightweight method

## Abstract

Keyword spotting (KWS) systems based on Spike Neural Networks (SNNs) offer a promising solution for always-on voice interfaces. However, achieving a favorable trade-off between computational footprint and recognition accuracy remains challenging for resource-constrained edge devices. This paper proposes a lightweight convolutional spiking neural network (CSNN) for KWS that combines a streamable Mel-to-Spike encoder, a convolutional spiking feature extractor, and a delay-aware classification module that uses learnable synaptic delays. The proposed encoder adopts streaming frame-by-frame encoding to convert speech features into sparse spike trains, while the delay-aware classifier jointly optimizes synaptic weights and temporal delays for enhanced spatiotemporal evidence aggregation. Experiments on the Google Speech Commands V1 and V2 (GSC-V1 and GSC-V2), Heidelberg Digits (HD), and Chinese Mandarin Keyword (CMK) datasets show mean test accuracies of 94.37%, 92.87%, 99.10%, and 95.60%, respectively. The proposed method uses only 64.05 K and 68.14 K learnable parameters for the 12-class and 20-class classification, while maintaining strong robustness to additive noise. These results indicate that the proposed CSNN achieves a favorable algorithm-level balance among accuracy, compactness, and noise robustness for KWS.

## 1. Introduction

Keyword spotting (KWS) is a key enabler for always-on voice interfaces on edge devices such as smartphones, smart speakers, and wearable systems [1,2]. Although conventional KWS systems based on artificial neural networks (ANNs) can achieve high recognition accuracy [3,4,5], their dense computation and frequent memory access make long-term deployment on resource-constrained hardware challenging [6,7]. Recently, spiking neural networks (SNNs) have been regarded as the third generation of neural network architectures [8,9,10], which process information through discrete spike events and activate computation only when informative events occur [11]. This event-driven property has motivated many studies to investigate SNNs as compact models with sparse computation for edge-oriented KWS [12].

Recent studies have begun exploring the use of spiking neural networks (SNNs) for keyword spotting and have shown that event-driven models can achieve promising recognition performance [1,13]. One line of research focuses on spike-based speech benchmarks with biologically inspired auditory encoding. Cramer et al. [14] introduced the Spiking Heidelberg Digits (SHD) and Spiking Speech Commands (SSC) datasets and demonstrated the importance of spike timing information for speech classification performance. They achieved 83.2% accuracy on SSC and 50.9% on SHD using a recurrent SNN. Building on these benchmarks, Baronig et al. [15] improved performance to 95.81% on SHD and 80.44% on SSC, while Sun et al. [16] further increased accuracy on SHD to 96.26% with a parameter-free attention mechanism. Although these efforts have advanced spike-based speech recognition, their main emphasis is often on benchmark performance and temporal modeling. In addition, biologically inspired cochlear encoding usually requires substantial buffering for spike-generation pipelines, which may not be fast enough for real-time operation even on a high-end workstation [17]. This may complicate streaming inference and the implementation of practical edge-oriented KWS systems.

Another line of research investigates end-to-end SNN architectures for keyword spotting. Auge et al. [7] proposed an end-to-end recurrent SNN with resonant input neurons, reaching 86.7% accuracy on GSC-V1 under constrained hardware. Yang et al. [18] further introduced a deep residual SNN with a 1D convolutional frontend and an enhanced progressive tandem learning algorithm, achieving 92.2% and 92.9% accuracy on GSC-V1 and GSC-V2, respectively, with 86.5 K learnable parameters for portable-device scenarios. Recently, Wang et al. [12] proposed a lightweight SNN-KWS model based on Global-Local Spiking Convolution and Bottleneck-PLIF modules, achieving 93.0% on GSC-V1 and 94.4% on GSC-V2 with 70.1 K learnable parameters. Stewart et al. [17] introduced the Speech2Spikes pipeline with an SDFT-based log-Mel frontend, achieving 88.5% accuracy on GSC-V2 and 98.3% on HD with 160 k learnable parameters. These studies demonstrate the potential of SNNs to perform KWS in resource-constrained environments, but practical edge application still involves key trade-offs. Recurrent and deep SNNs improve temporal modeling but add structural complexity, while lightweight convolutional SNNs reduce parameter cost but often provide limited temporal aggregation through simple readout layers. Streamable frontends have been explored, but their integration with compact spiking backbones and efficient temporal classifiers remains limited.

To address this issue, a lightweight and innovative CSNN for KWS is proposed that combines a streamable log-Mel-based spike encoder, a convolutional spiking feature extractor, and a delay-aware fully connected (DAFC) classifier. The proposed encoder converts continuous-valued features into sparse spike trains frame by frame, avoiding reliance on pre-generated and buffered cochlear spikes. The spiking convolution then extracts spectrotemporal features, while the DAFC classifier module introduces learnable synaptic delays via one-dimensional dilated convolution with learnable spacings (1D-DCLS) [19] to improve temporal evidence aggregation at low parameter cost. The proposed CSNN with synaptic delays achieves competitive classification performance with a compact parameter budget across all evaluated datasets. We further evaluate its analytical computational complexity, memory footprint, and robustness under SNR-controlled additive noise, providing a model-level assessment for edge-oriented KWS.

The contribution of this study can be summarized as follows:

- The proposed speech encoding strategy for KWS converts log-Mel features into sparse spike trains in a streamable manner, reducing reliance on buffered spike preprocessing and supporting an edge-oriented model design.
- The paper introduces a delay-aware fully connected classification method based on 1D-DCLS, which incorporates learnable synaptic delays into the classifier module without substantially increasing the model size, thereby enabling more effective spatiotemporal feature integration and coincidence detection over temporal intervals.
- The proposed method achieves competitive recognition accuracy and noise robustness across four evaluated datasets while maintaining a compact learnable-parameter budget, indicating a favorable algorithm-level balance among accuracy, compactness, and robustness.

The rest of this paper is organized as follows. Section 2 introduces the proposed lightweight CSNN framework for keyword spotting. Section 3 describes the datasets, experimental setup, and ablation studies. Section 4 presents the experimental results, a comparative analysis, and a discussion of memory and computation complexity. Finally, Section 5 concludes the paper and outlines future research directions.

## 2. Methodology

**Figure 1:**
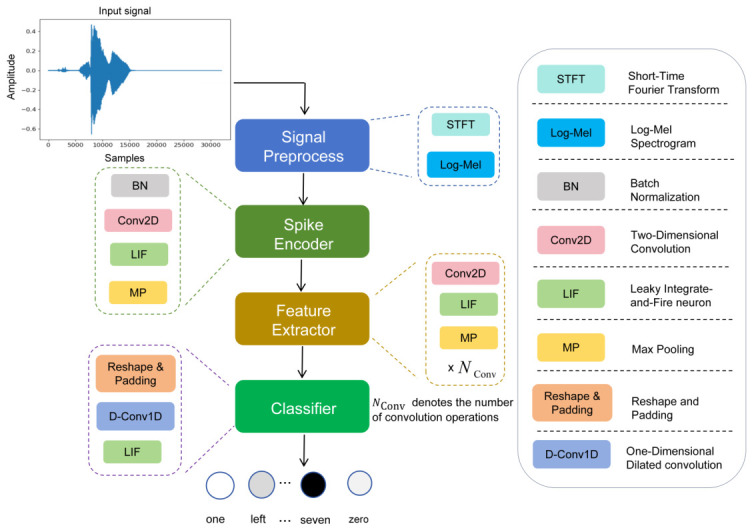
Overall framework of the proposed CSNN method for KWS. The pipeline consists of signal preprocessing, spike encoding, spiking convolutional feature extraction, and a DAFC-based classifier. Conv2D, LIF, MP, and D-Conv1D in the figure denote two-dimensional convolution, leaky integrate-and-fire activation, max pooling, and one-dimensional dilated convolution with learnable synaptic delays, respectively.

The overall framework of the proposed lightweight CSNN for keyword spotting is shown in Figure 1. It consists of a streamable LogMel-to-spike encoder, a spiking convolutional feature extractor, and a delay-aware fully connected classifier. The input waveform is first transformed into log-Mel features, then encoded into sparse spike representations, processed by a spiking convolutional feature extractor, and finally classified by a delay-aware classifier. The following subsections describe these components in detail.

### 2.1. Signal Preprocess Module

To obtain a compact and streamable acoustic representation for keyword spotting, we use a log-Mel-based preprocessing frontend composed of three stages. First, the input digital audio sequence is transformed into the frequency domain using the Sliding Discrete Fourier Transform (SDFT), which updates spectral coefficients incrementally and reuses intermediate spectral calculations [20]. This property makes SDFT particularly suitable for streaming and low-latency speech processing on edge devices [17]. Thereafter, the spectrum is projected onto Mel-scale filter banks to reduce spectral redundancy and to retain the perceptually relevant speech information [13,21]. Finally, the Mel-band energies are compressed logarithmically [22] to obtain the final log-Mel representation, which is fed to the subsequent spike encoder.

In the experiments, all input audio is resampled to 16 kHz. Each utterance is converted into a log-Mel representation using fixed Mel filter banks. The parameters are fixed as shown in Table 1 to ensure the resulting time–frequency representation is standardized to 101×64 before being fed into the spike encoder. For numerical stability, the logarithmic compression is implemented as log(x+ϵ), where ϵ is a small constant to avoid singular values.

### 2.2. Spike Encoding Module

**Figure 2:**
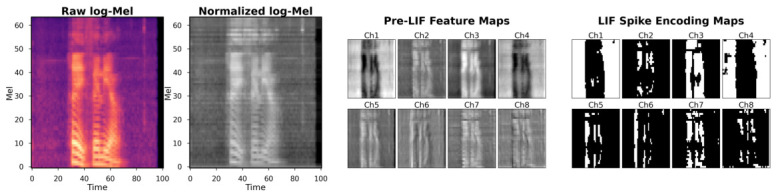
Visualization of the LogMel-to-spike encoding process. The LogMel spectrogram is normalized into a log-Mel feature map and then projected by the spike encoder into eight sparse spike channels through Conv2D and LIF activation. The resulting channel-wise spike maps provide event-driven representations for the subsequent spiking convolutional backbone.

Rather than relying on a handcrafted auditory spike conversion scheme, the spike encoding module in the proposed method converts the input log-Mel representation into sparse spike trains through convolution. Specifically, batch normalization is first applied to reduce amplitude variation and stabilize feature propagation. Then a 5×5 two-dimensional convolution without padding projects the normalized feature into 8 channels, producing feature maps of size 97×60×8, as shown in Figure 2. These features are then unfolded over the simulation step of length *T* and integrated by LIF neurons to generate sparse binary spike trains. In this way, the encoder serves as a trainable speech-to-spike interface, producing compact event-driven representations while preserving discriminative acoustic features for the downstream spiking backbone.

### 2.3. LIF Neuron

**Figure 3:**
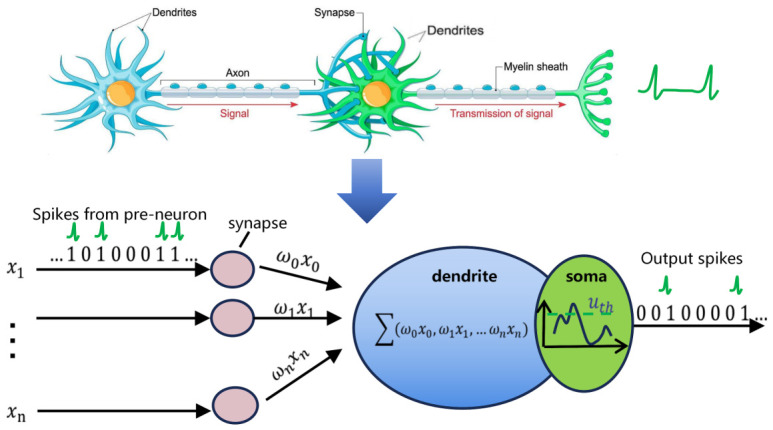
Biological motivation and discrete abstraction of the LIF neuron. The upper part shows biological signal transmission through dendrites, synapses, and axons, while the lower part illustrates the LIF neuron model, where weighted presynaptic spikes are integrated and emitted as output spikes once the membrane potential exceeds $U_{thr}$.

The leaky integrate-and-fire (LIF) neuron is used as the basic computational unit of the proposed method for its simplicity [23], biological interpretability, and efficient implementation [24,25]. As illustrated in Figure 3, a LIF neuron integrates incoming spike-driven current into its membrane potential, exhibits temporal leakage, and emits an output spike when the membrane potential exceeds a predefined threshold [26]. This mechanism mimics biological neurons and provides a compact, effective way to model temporal dynamics in spiking neural networks [27].

The membrane dynamics of an LIF neuron in discrete time can be expressed as:

(1) $$U_{i}^{l}\left(t\right)=\beta *U_{i}^{l}\left(t-1\right)+I_{i}^{l}\left(t\right)$$

where $U_{i}^{l}\left(t\right)$ denotes the membrane potential of neuron *i* in layer *l* at simulation step *t*, $I_{i}^{l}\left(t\right)$ is the synaptic input current, and β∈[0,1] is the membrane decay factor. The decay factor β is the discrete-time counterpart of the continuous membrane time constant $\tau_{m}$. For example, under exponential discretization, they can be related by $\beta =\exp(-\Delta t/\tau_{m})$.

The synaptic input current is computed as:

(2) $$I_{i}^{l}\left(t\right)=\sum_{j}\omega_{ij}^{l}*S_{j}^{l-1}\left(t\right)$$

where $\omega_{ij}^{l}$ is the synaptic weight from presynaptic neuron *j* to postsynaptic neuron *i*, and $S_{j}^{l-1}\left(t\right)$ denotes the incoming spike train from layer l−1.

A neuron generates a spike when its membrane potential exceeds the predefined firing threshold $U_{thr}$,

(3) $$S_{t}^{l}=f_{s}\left(U_{i}^{l}\left(t\right)\right)=\delta \left(t-t_{f}\right)=\left\{\begin{array}{cc} 1, & ifU_{i}^{l}\left(t\right)\geq U_{thr};\\ 0, & ifU_{i}^{l}\left(t\right)<U_{thr}. \end{array}\right.$$

where $f_{s}\left(\cdot \right)$ denotes the spike generation function and $t_{f}$ indicates the firing time.

In the proposed method, the membrane decay factor is fixed to β=0.5, and the firing threshold is set to $U_{\mathrm{thr}}$=1.0 for all LIF neurons. This means that half of the previous membrane potential is retained at each simulation step before integrating the new synaptic input. The value β=0.5 was used as a stable default setting in the snnTorch-based implementation and was kept fixed across all datasets and ablation experiments. After a spike is emitted, the membrane potential is reset to zero according to Equation (3).

### 2.4. Spiking Convolutional Feature Extraction Module

**Figure 4:**
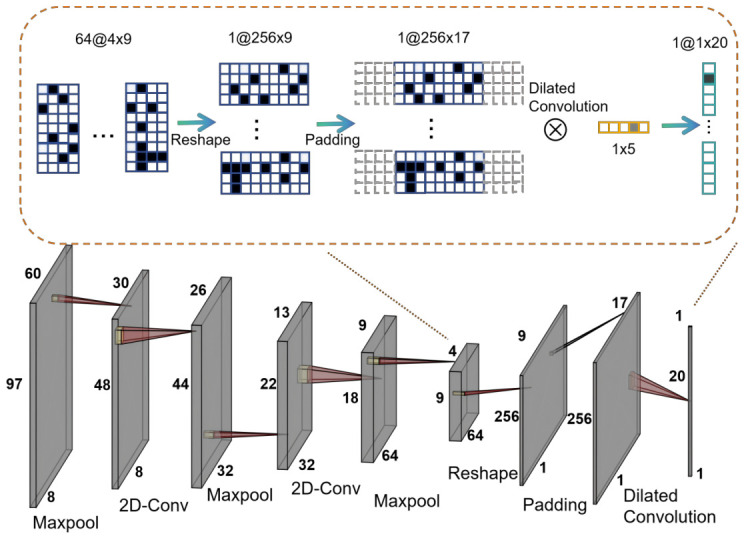
Tensor-shape evolution of the proposed CSNN-DAFC architecture. The lower path shows the shape changes across the spiking convolutional backbone. The upper inset details the DAFC, where the spike tensor is reshaped into a 1×256×9 latent temporal representation, padded to 1×256×17, and projected to the class space by a 1D-DCLS convolution, which uses a bank of $256\times n_{\mathrm{class}}$ learnable-delay temporal kernels with size 1×5.

The spiking convolutional feature extractor processes the encoded spike trains to produce compact spectrotemporal representations for classification. The feature extractor is composed of a sequence of stacked convolutional layers, LIF activation, and max-pooling operations as illustrated in Figure 4 and Table 2. This hierarchical design incrementally increases the receptive field through convolution while also reducing the spatial dimensions of intermediate spike representations through max-pooling, enabling the network to capture distinctive acoustic patterns while maintaining a compact parameter budget in an event-driven manner. 

As shown in Figure 4, the first max-pooling layer with a 2×2 kernel reduces the encoder output from 97×60×8 to 48×30×8. The resulting spike representation is then processed by two additional convolutional blocks with 5×5 kernels and 32 and 64 output channels, respectively. Each convolutional stage is followed by LIF-based spike generation and max pooling, enabling the network to extract higher-level spectrotemporal features while gradually compressing the representation. After the last max-pooling layer, the feature tensor is reduced to a compact 9×4×64 representation. This tensor is reorganized into a 256×9 latent temporal sequence by flattening the channel-frequency dimensions while preserving the compressed temporal axis, and is then fed into the DAFC classifier for delay-aware temporal aggregation.

The learnable-parameter counts in Table 2 include convolutional weights and bias, as well as all trainable synaptic weights and synaptic delay parameters in the DAFC classifier. Batch normalization, LIF neurons, padding, max pooling, and temporal aggregation do not introduce learnable parameters. Accordingly, the total learnable parameter count is 64.05 K for the 12-class setting, and 68.14 K for the 20-class setting.

### 2.5. Delay-Aware Fully Connected Classification Module

In biological neural systems, spike transmission from a presynaptic neuron to a postsynaptic neuron is often associated with temporal delays [28]. Such synaptic delays can be interpreted in discrete time as shifts in the effective temporal positions of synaptic responses. Motivated by this observation, and the connection between synaptic delays and one-dimensional temporal convolutions [19], a delay-aware fully connected (DAFC) classification module is integrated into the method. Unlike a conventional fully connected (CFC) layer that directly maps features to class logits, the proposed DAFC module incorporates learnable temporal offsets into each feature-to-class connection through 1D-DCLS.

**Figure 5:**
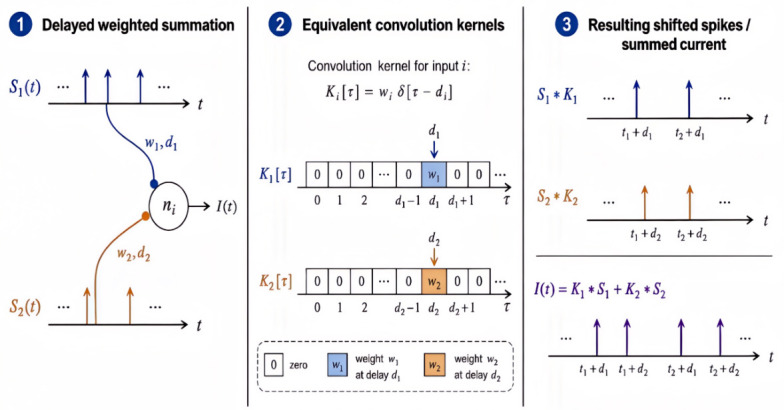
Delay-aware summation interpreted as one-dimensional temporal convolution. Each presynaptic connection is represented by a sparse temporal kernel whose non-zero coefficient encodes the synaptic weight at the corresponding delay position. Convolving the input spike train with this kernel shifts and weights the spikes, and the postsynaptic current is obtained by summing all delayed contributions.

The key idea of the proposed DAFC module is that each connection from a latent feature unit to an output class can contribute at a learnable delayed temporal position rather than only at the aligned time instant. Figure 5 illustrates how this delayed weighted summation can be equivalently represented by sparse one-dimensional temporal kernels.

In the CFC layer, the postsynaptic response is typically computed as a temporally aligned weighted sum. In contrast, the proposed DAFC module introduces a learnable delay for each connection, so that the input current of neuron *i* in layer *l* is redefined from Equation (2) as follows:

(4) $$I_{i}^{l}\left(t\right)=\sum_{j=1}^{N}\left(\omega_{ij}^{l}S_{j}^{l-1}\left[t-d_{ij}^{l}\right]\right)=\sum_{j=1}^{N}\left(k_{ij}^{l}*S_{j}^{l-1}\right),$$

Here $S_{j}^{l-1}$ denotes the padded latent temporal sequence for feature unit *j* in layer l−1, and $k_{ij}^{l}$ denotes the 1D-DCLS kernel for the connection from presynaptic feature unit *j* to postsynaptic neuron *i*. Each kernel is parameterized by a learnable synaptic weight $\omega_{ij}^{l}$ and a learnable delay $d_{ij}^{l}$, with the delay determining the kernel’s effective non-zero temporal location. In the discrete case, the kernel can be expressed as follows for all n∈[0,…,M−1]:

(5) $$k_{ij}^{l}\left(n\right)=\left\{\begin{array}{cc} \omega_{ij}^{l}, & ifn=M-d_{ij}^{l}-1;\\ 0, & \mathrm{otherwise}. \end{array}\right.$$

where *M* denotes the kernel support length, equal to the maximum delay range plus one. Under this formulation, each feature-to-class connection contributes to the postsynaptic response at a learnable temporal offset, rather than only at the current aligned position.

During training, the discrete kernel location is relaxed into a differentiable form using 1D-DCLS. Specifically, the effective kernel is modeled as a Gaussian interpolation centered at $M-d_{ij}^{l}-1$, where $d_{ij}^{l}\in \left[0,\ldots ,M-1\right]$ denotes the learnable temporal position and $\sigma_{ij}^{l}$ controls the interpolation width:

(6) $$k_{ij}^{l}\left[n\right]=\frac{\omega_{ij}^{l}}{c}\exp\left(-\frac{1}{2}{\left(\frac{n-M+d_{ij}^{l}+1}{\sigma_{ij}^{l}}\right)}^{2}\right),$$

where *c* is a normalization factor defined as:

(7) $$c=\epsilon +\sum_{n=0}^{M-1}\exp\left(-\frac{1}{2}{\left(\frac{n-M+d_{ij}^{l}+1}{\sigma_{ij}^{l}}\right)}^{2}\right),$$

Here $\epsilon =10^{-7}$ is a small constant introduced for numerical stability. The parameter $\sigma_{ij}^{l}$ controls the interpolation smoothness and enables gradient-based optimization of the temporal position during training. After each optimization step, the learned positions are clamped to the valid interval [0,M−1]. During inference, the continuous positions are discretized to the nearest valid temporal index, yielding sparse delay-aware kernels with a single effective non-zero location.

As shown in Figure 4, the compact features produced by the spiking convolutional backbone are rearranged into a 256×9 latent temporal representation at each simulation step, where 256 denotes the latent feature dimension and 9 denotes the compressed temporal length. To support delay-aware aggregation near sequence boundaries, zero-padding is applied to both sides of the compressed temporal axis. In the implementation, the DCLS kernel size is set to M=5, and the padding width on each side is set to ΔP=M−1=4. Thus, the 256×9 latent representation is expanded to 256×17, allowing the DAFC readout to select delayed evidence near boundary positions without discarding edge information.

The padded latent representation is then projected into the class space via the 1D-DCLS computation, with $n_{\mathrm{class}}=12$ or 20 depending on the task. Each feature-to-class connection has a learnable synaptic weight and a learnable temporal delay, enabling local delay-aware aggregation along the latent temporal axis. The resulting class currents are passed through the output LIF layer, and the final keyword prediction is obtained by spike-count readout over all simulation steps.

## 3. Experiments

To comprehensively evaluate the proposed method, experiments were conducted on four datasets. The evaluation covers three aspects: ablation studies of key model settings, including the simulation time step *T* and the surrogate gradient function, classifier-level comparisons between the proposed DAFC and alternative classifier variants, and robustness assessment under SNR-controlled additive noise.

### 3.1. Datasets

**Figure 6:**
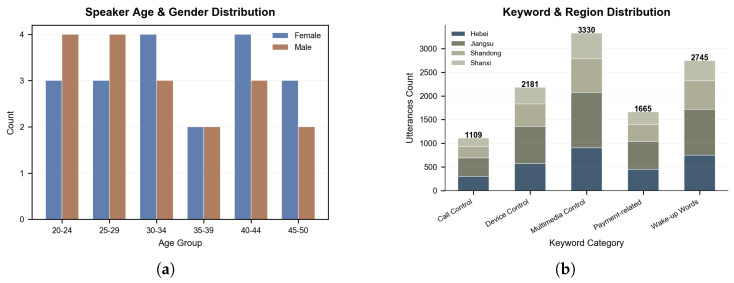
Overview of the Chinese Mandarin Keyword (CMK) dataset. The dataset contains 11,030 Mandarin keyword utterances collected for voice interaction applications and spans 20 keyword classes. (**a**) Distribution of speakers by age group and gender. The dataset includes both male and female speakers across six age groups with relatively balanced demographic coverage. (**b**) Distribution of utterances across geographic regions and keyword categories, including wake-up, device-control, multimedia-control, payment-related, and call-control commands.

The proposed method is evaluated on four datasets, including public English and German keyword benchmarks and a self-collected Mandarin keyword corpus:

- **GSC-V1**: Google Speech Commands V1 is a widely used benchmark for keyword spotting [29,30]. It contains 64,727 one-second utterances of 30 short English words spoken by 1881 speakers and recorded at 16 kHz. In this work, the dataset is used in the 12-class KWS setting (10 keywords, “Unknown” and “Silence”).
- **GSC-V2**: Google Speech Commands V2 [30,31] is an extended version of the GSC-V1. It contains 105,829 one-second utterances from 2618 speakers across 35 spoken word classes, recorded at 16 kHz. Similar to GSC-V1, this dataset is evaluated in the 12-class KWS setting.
- **HD**: Heidelberg Digits [14] is a spoken-digit benchmark commonly used to evaluate spiking neural networks. It contains 10,420 recordings of English and German digits from 12 speakers, originally sampled at 48 kHz, and is treated as a 20-class classification task in this work.
- **CMK**: To evaluate the proposed method in Mandarin voice-interaction scenarios, we collected a Chinese Mandarin Keyword (CMK) dataset recorded in indoor environments at 48 kHz and 16-bit resolution. The dataset contains 11,030 utterances from 36 speakers, covering both genders and four geographic regions in China. The CMK dataset is organized with speaker identifiers, keyword labels, gender, age-group, and geographic-region metadata. Figure 6a and Figure 6b summarize the speaker demographics and keyword-category distribution across geographic regions, respectively.

### 3.2. Experimental Configuration

All experiments were conducted on a workstation equipped with an Intel Xeon W-2223 CPU, 32 GB of RAM, and an NVIDIA GeForce RTX 3090 GPU. The proposed method was implemented in PyTorch using the snnTorch framework [32], together with auxiliary libraries for data processing and spike-based training.

All audio samples were resampled to 16 kHz before feature extraction. For GSC-V1 and GSC-V2, a random 70%/10%/20% split was used to construct the training, validation, and test subsets, and an additional evaluation was conducted using the official 80%/10%/10% split setting. For HD, both a random 70%/10%/20% split and the official train/test partition were evaluated. For CMK, both a random 70%/10%/20% split and a strict speaker-independent 70%/10%/20% split were constructed. In the random split, utterances from the same speaker may appear in different subsets, whereas the speaker-independent split excludes test speakers from training and validation.

During training, the batch size was set to 256. The model parameters were tuned using Adam, the learning rate for weights was set to 0.001, and the learning rate of delay parameters in the DAFC module was set to 0.1. Because spike generation is non-differentiable, surrogate gradients are required during backpropagation [33]. The method was trained for up to 500 epochs using the mean squared error (MSE) spike-count loss, with early stopping when the validation loss did not improve for 20 consecutive epochs. For a fair comparison, each split setting was trained ten times with different random seeds, and the same optimization settings were used in all experiments.

### 3.3. Training and Ablation Study

**Figure 7:**
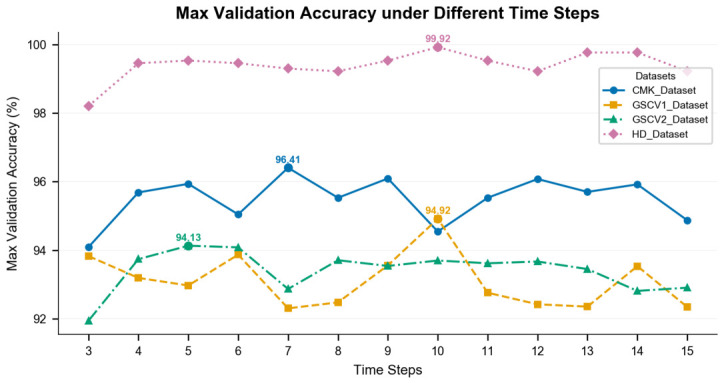
Validation accuracy of the proposed DAFC-based CSNN as a function of the simulation time step *T* across four datasets. Increasing *T* generally improves performance up to a task-dependent operating point, beyond which gains become marginal or may even degrade.

For spike-driven KWS systems intended for resource-constrained edge application, the simulation time step *T* is a critical hyperparameter. It determines the temporal unfolding length of the CSNN and directly affects both recognition performance and computational overhead. We therefore evaluate the validation accuracy of the proposed method across different values of *T* on all four datasets to identify a suitable trade-off between recognition accuracy and computational efficiency. As shown in Figure 7, the proposed method achieved the best validation accuracy on the four datasets with $T_{cmk}=7$, $T_{gsc-v1}=10$, $T_{gsc-v2}=5$, and $T_{hd}=10$, respectively.

**Figure 8:**
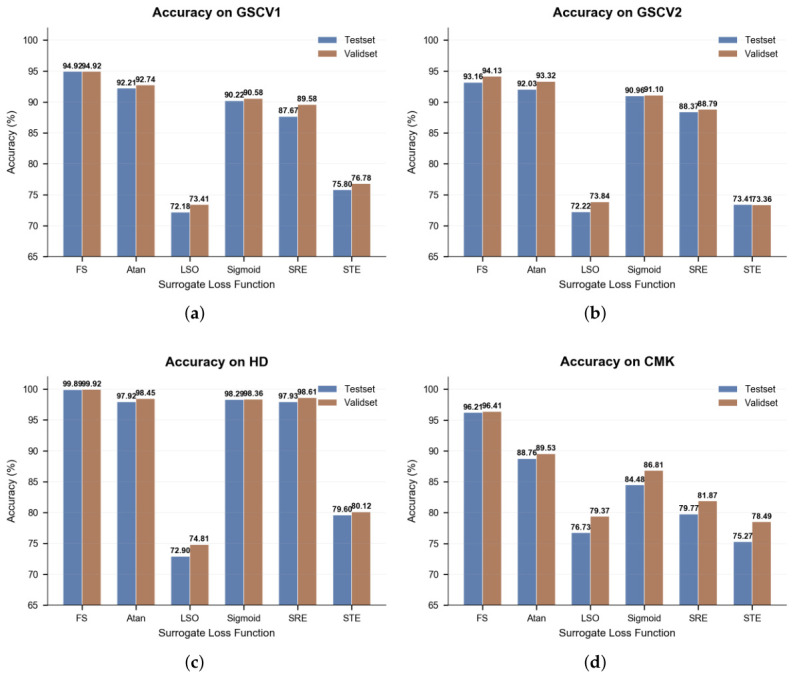
Comparison of surrogate gradient functions across four datasets: (**a**) GSC-V1, (**b**) GSC-V2, (**c**) HD, and (**d**) CMK. For each surrogate function, both validation and test accuracies are reported.

To assess the proposed model’s sensitivity to the choice of surrogate function, six representative surrogate gradients: Fast Sigmoid (FS), Atan, Sigmoid, Spike Rate Escape (SRE), Straight-Through Estimator (STE), and Leaky Spike Operator (LSO) were compared. The comparison was conducted on four datasets, and the resulting validation and test accuracies are summarized in Figure 8. The Fast Sigmoid (FS) surrogate performs the best among the four datasets and is a consistently good choice for validation and test accuracy. Atan yields competitive and relatively stable results on datasets. In contrast, LSO and STE offer much lower recognition accuracy, suggesting they are not suitable for optimizing the proposed method. Sigmoid and SRE have a medium performance. Overall, these results indicate that the choice of surrogate gradient significantly impacts training effectiveness and final recognition accuracy [34], and FS is the most favorable option for the proposed DAFC-based CSNN.

**Figure 9:**
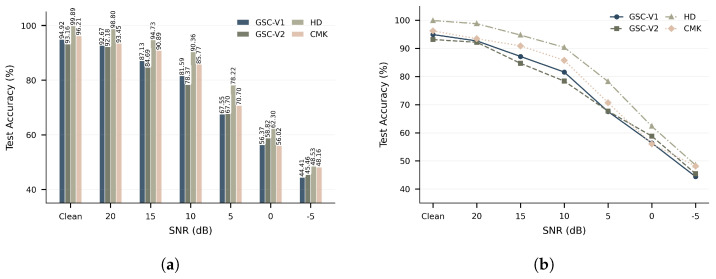
Test accuracy of the proposed CSNN method under SNR-controlled additive Gaussian noise across four datasets. The bar plot in (**a**) compares accuracies across four datasets at each SNR level, and the line plot in (**b**) shows the accuracy degradation trends as SNR decreases. The evaluated conditions were clean, 20, 15, 10, 5, 0, and −5 dB, where “clean” denotes the original test waveform without added noise. (**a**) Test accuracy at different SNR levels. (**b**) Accuracy degradation trends with decreasing SNR.

To evaluate the robustness of the proposed lightweight method, we used SNR-controlled additive white Gaussian noise (AWGN) applied only during inference [16]. For each clean utterance *x* with average power $P_{x}$, the corrupted utterance was generated by adding zero-mean Gaussian noise scaled according to the target SNR: $\tilde{x}=x+\sqrt{P_{x}/10^{\mathrm{SNR}/10}}z$, where z∼N(0,1). As shown in Figure 9, the recognition accuracy gradually decreases for all four datasets with increasing noise, because stronger corruption reduces the separability of acoustic patterns. Nevertheless, the proposed DAFC-based CSNN maintains relatively stable performance over a wide range of noise levels, indicating that the learned spike-based representations remain discriminative under moderate acoustic degradation.

To better isolate the contribution of the DAFC classifier, we further compare four classifier variants as summarized in Table 3, using the same preprocessing frontend, spiking convolutional backbone, and SNR-controlled evaluation procedure. Only the classifier module is changed in this comparison, all classifier variants use the same spike feature-map tensor of size 64×9×4 produced by the extractor.

**Figure 10:**
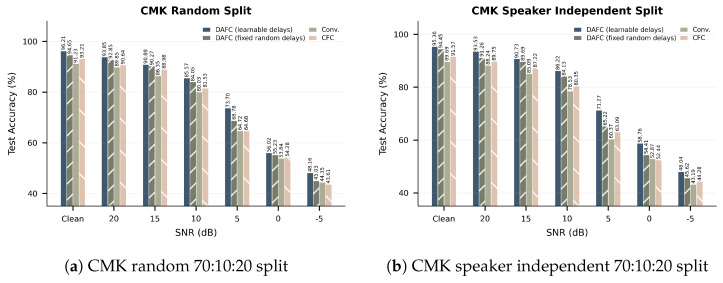
Classifier ablation on the CMK dataset under different SNR. The comparison includes the proposed DAFC with learnable delays, DAFC with fixed random delays, a temporal convolutional classifier, and CFC. The results are shown under (**a**) the random 70:10:20 split and (**b**) the speaker-independent 70:10:20 split.

As shown in Figure 10, comparative experiments are conducted on the CMK dataset under both random split and speaker-independent split settings. The DAFC with learnable delays shows competitive robustness among the evaluated classifier variants in both split settings. In the random split setting, DAFC with learnable delays attains the peak accuracy of 96.21% on clean speech and remains at 48.16% at −5 dB, whereas the DAFC with fixed random delays, temporal convolution, and CFC variants obtain 94.65%, 91.23%, and 93.21% on clean speech, and 45.03%, 44.35%, and 43.61% at −5 dB, respectively. In the speaker-independent split setting, the DAFC with learnable delays also provides the highest accuracy over the full SNR range, including 95.36% on clean speech and 48.04% at −5 dB. The fixed-random-delay DAFC reduces the performance gap relative to the learnable-delay DAFC, indicating that temporal offsets contribute to the classifier. Nevertheless, its consistently lower accuracy suggests that learnable delay positions are preferable. The temporal convolutional classifier provides a convolution-based readout baseline, but it does not achieve comparable accuracy to the DAFC and CFC under all tested SNR conditions. This comparison suggests that making the delay parameters learnable may be beneficial for the DAFC module.

## 4. Results and Discussion

**Figure 11:**
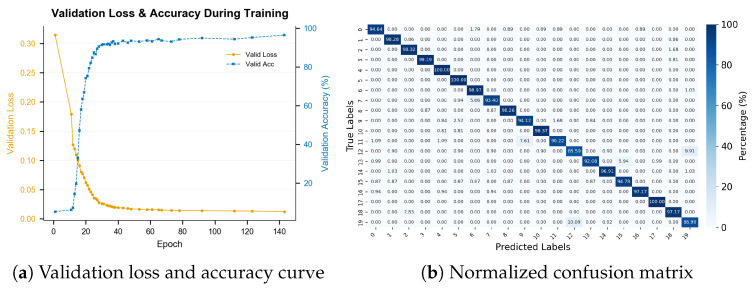
Training dynamics and class-wise recognition performance of the proposed CSNN on the CMK dataset. (**a**) Validation loss and accuracy over training epochs. (**b**) Normalized confusion matrix for the test set.

In our experiments, the training dynamics and class-wise recognition behavior of the proposed CSNN with synaptic delays model on the CMK dataset are shown in Figure 11. As shown in Figure 11a, the validation loss decreases rapidly in the early stages of training, while the validation accuracy rises sharply and stabilizes after approximately 30 epochs. The best validation accuracy reaches 96.41% at epoch 143, indicating that the proposed CSNN can be optimized stably and converges to a high-performance operating point without severe oscillation or overfitting. The normalized confusion matrix in Figure 11b further shows that the model achieves strong discrimination across most keyword classes, with most diagonal entries remaining at high recognition rates. Most categories exceed 95% true positive rate, and several reach 100%, while a limited number of classes show moderate confusion with acoustically similar categories.

To analyze the proposed method and prior SNN-based keyword spotting studies, Table 4 summarizes representative previously reported results together with the available split setting. We trained and evaluated the proposed method ten times with different random seeds under all split settings. For GSC-V1, GSC-V2 and HD, the official split setting and the random 70%/10%/20% split setting were applied. For CMK, results are reported under the random split and a more restrictive speaker-independent evaluation. The random split partitions utterances into training, validation, and test subsets, so utterances from the same speaker may appear in different subsets, whereas the speaker-independent split separates speakers and excludes test speakers from training and validation. The latter setting provides a more stringent evaluation of cross-speaker generalization. For CMK, we reproduced two representative delay-based SNN baselines, SNN with Delay and DCLS-Delays, under the same split setting used by the proposed method.

As shown in Table 4, the proposed CSNN provides a competitive trade-off between recognition accuracy and learnable-parameter count across the evaluated settings. Since the compared methods differ in datasets splits, input representations, preprocessing pipelines, and data augmentation strategies, Table 4 should be interpreted as a literature-level comparison rather than a strictly controlled comparison under identical conditions.

To complement the recognition results, this section provides an analytical discussion of inference complexity and memory footprint. Memory cost is a first-order design constraint for edge-oriented KWS models [42]. In the proposed method, the total learnable parameter count is only 64.05 K in the 12-class setting and 68.14 K in the 20-class setting, corresponding to 64.05 KB and 68.14 KB under 8-bit fixed-point quantization. In addition to storing the weight parameters, the dominant dynamic memory usage comes from neuron states. According to Table 2, 93,556 membrane states require about 182.7 KB under 16-bit representation. By contrast, the storage required for spike-domain intermediate tensors is much smaller, since binary spike maps can be bit-packed. Summing the principal spike tensors in Table 2 yields about 116,512 bits, approximately 14.2 KB for one simulation step. A full 16-bit buffer for the 101×64 log-Mel representation requires about 12.6 KB, but this upper-bound estimate can be reduced through streaming feature processing with local buffering [43]. Furthermore, intermediate spike buffers can be partially reused across adjacent pipeline stages via ping-pong buffering or BRAM bank reuse. Therefore, the reported 270–300 KB memory footprint should be understood as an analytical storage estimates under the stated bit-width and buffering assumptions, as shown in Table 5, dominated mainly by weight-parameter storage, neuron-state memory, preprocess frontend buffers, and spike buffering.

The proposed method exhibits a mixed dense and spike-driven computation pattern. In contrast, the acoustic pre-processing frontend and the first encoder layer still process continuous-valued log-Mel features. The spike convolutional backbone and the delay-aware classifier can be executed event-drivenly after spike encoding. The Multiply-Add Calculations (MAC) values reported below correspond to dense-equivalent inference complexity rather than the exact arithmetic executed in a spike-driven FPGA implementation. According to Table 2, the first encoder convolution requires

(8) $${\mathrm{MAC}}_{\mathrm{Conv}\_L1}=97\times 60\times 8\times 5\times 5\times 1=1,164,000,$$

which is incurred once per utterance and cannot be replaced by Add Calculations (AC) operations because the input remains dense. The two deeper spiking convolutional layers have dense-equivalent costs of

(9) $${\mathrm{MAC}}_{\mathrm{Conv}\_L2}^{\mathrm{dense}}=7,321,600,\;{\mathrm{MAC}}_{\mathrm{Conv}\_L3}^{\mathrm{dense}}=8,294,400,$$

per simulation step. For the DAFC classifier, the dense-equivalent inference cost is

(10) $${\mathrm{MAC}}_{\mathrm{DAFC}}^{\mathrm{dense}}=L_{out}\;n_{\mathrm{class}}\;M\;C_{in},$$

where $L_{out}=13$, M=5, and $C_{in}=256$, resulting in 199,680 operations per step for the 12-class setting and 332,800 for the 20-class setting.

Therefore, the total dense-equivalent MAC count per utterance is

(11) $${\mathrm{MAC}}_{12}^{\mathrm{dense}}=1,164,000+T\times \left(7,321,600+8,294,400+199,680\right),$$

for the 12-class setting, and

(12) $${\mathrm{MAC}}_{20}^{\mathrm{dense}}=1,164,000+T\times \left(7,321,600+8,294,400+332,800\right).$$

Using the selected simulation steps, the corresponding values are approximately 159.32 M MACs for GSC-V1, 80.24 M for GSC-V2, 160.65 M for HD, and 112.81 M for CMK.

However, during spike-driven inference, the computations in Conv_L2, Conv_L3, and the discretized DAFC layer no longer need to be performed as full MAC operations. Because the spike input is binary, the synaptic product w·s reduces to a conditional accumulation: when s=1, the weight is added to the target neuron; when s=0, no operation is performed [44]. Let $\rho_{1}$, $\rho_{2}$, and $\rho_{3}$ denote the average spike activity ratios for Conv_L2, Conv_L3, and the classifier input, respectively. Then the practical AC count of the proposed model can be approximated by

(13) $${\mathrm{AC}}_{12}=T\times \left(\rho_{1}\cdot 7,321,600+\rho_{2}\cdot 8,294,400+\rho_{3}\cdot 39,936\right),$$

for the 12-class setting, and

(14) $${\mathrm{AC}}_{20}=T\times \left(\rho_{1}\cdot 7,321,600+\rho_{2}\cdot 8,294,400+\rho_{3}\cdot 66,560\right),$$

for the 20-class setting, where 39,936 and 66,560 denote the estimated accumulation costs of the discretized DAFC readout. These estimates assume that the learned delays are converted into fixed delay indices during inference.

The spike activity ratios in Equations (13) and (14) were obtained by instrumenting the trained model during inference. For each dataset, the best validation checkpoint was evaluated on the test set during inference, the spike tensors input to Conv_L2, Conv_L3, and the DAFC classifier were recorded at each simulation step, and their emitted spikes were accumulated over all test mini-batches.

For a test utterance *n*, the monitored spike activity ratio of layer *l* is defined as

(15) $$\rho_{l}^{\left(n\right)}=\frac{\sum_{t=1}^{T}\sum_{i}S_{l}^{\left(n\right)}\left(t,i\right)}{T|S_{l}|},$$

where $S_{l}^{\left(n\right)}\left(t,i\right)$ denotes the spike state of neuron *i* in layer *l* at simulation step *t*, *T* is the number of simulation steps, and $|S_{l}|$ is the number of neurons in that corresponding spike tensor for one utterance at one simulation step. The reported activity ratio was then obtained by averaging over all *N* test utterances:

(16) $${\bar{\rho }}_{l}=\frac{1}{N}\sum_{n=1}^{N}\rho_{l}^{\left(n\right)}=\frac{\sum_{n=1}^{N}\sum_{t=1}^{T}\sum_{i}S_{l}^{\left(n\right)}\left(t,i\right)}{NT|S_{l}|}.$$

All test utterances are padded or truncated to the same analysis length in each dataset, this is equivalent to dividing the total number of emitted spikes by the total number of monitored spike-tensor elements over the full test set. The resulting measured activity ratios are summarized in Table 6.

In addition to synaptic accumulation, the membrane states of LIF neurons must be updated at every simulation step. The number of neurons participating in temporal state updates is 93,556, so the state-update cost is

(17) StateUpdate=T×93,556.

Accordingly, the practical inference cost of the proposed model comprises a fixed dense MAC term from the frontend and encoder, sparse AC terms from the spike-driven backbone and the DAFC readout, and the neuron-state update cost:

(18) $${\mathrm{Cost}}_{\mathrm{inference}}={\mathrm{MAC}}_{\mathrm{dense}\_\mathrm{frontend}}+{\mathrm{AC}}_{\mathrm{spike}\_\mathrm{convolution}}+{\mathrm{AC}}_{\mathrm{DAFC}}+\mathrm{StateUpdate}.$$

**Figure 12:**
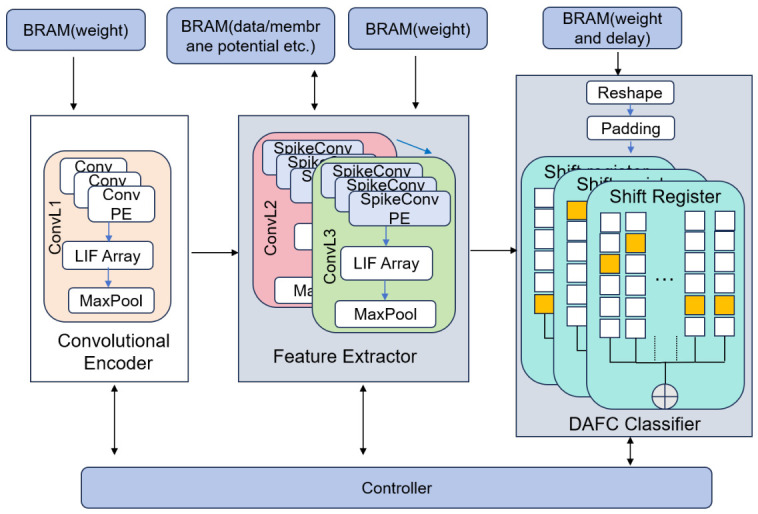
Conceptual FPGA-oriented architecture of the proposed CSNN-DAFC inference pipeline. The architecture consists of a dense convolutional encoder for continuous-valued log-Mel features, a spike-driven convolutional feature extractor for binary spike maps, and a DAFC classifier implemented through reshape, temporal padding, shift-register-based delay alignment, and class-wise accumulation. BRAM blocks store weights, delay indices, intermediate data, and membrane-potential states, while the global controller schedules simulation-step iteration, memory access, LIF state updates, DAFC readout, and spike-count voting.

Based on the above memory and computation analysis, Figure 12 illustrates a conceptual FPGA-oriented mapping of the proposed CSNN-DAFC inference pipeline. The first convolutional encoder corresponds to Conv_L1 and processes continuous-valued log-Mel features using dense convolution processing elements, which accounts for the fixed dense MAC term in Equation (8). The subsequent Conv_L2 and Conv_L3 feature-extraction stages operate on binary spike maps produced by LIF neurons and can therefore be mapped to spike-gated accumulation units, consistent with the sparse AC estimates in Equations (13) and (14).

The DAFC classifier is mapped to an inference-time delay-line readout. The final spike tensor of size 64×9×4 is reshaped into a 256×9 temporal sequence and padded before entering shift-register buffers. The learned delay locations are discretized into delay indices and stored together with DAFC weights in BRAM. During inference, these indices select temporal taps from the shift registers, and the selected binary spikes gate class-wise weight accumulation. Thus, the DAFC readout can be interpreted as delay-indexed sparse accumulation rather than a dense temporal convolution over all possible positions.

## 5. Conclusions

This paper presents a lightweight convolutional spiking neural network with learnable synaptic delays for keyword spotting that combines a streamable log-Mel-to-spike encoder, a spiking convolution backbone, and a delay-aware fully connected classifier based on 1D-DCLS. Unlike methods relying on buffered cochlear spike generation or heavy temporal modules, it adds learnable delays during classification to enhance temporal evidence aggregation while maintaining a compact structure.

Experimental results on GSC-V1, GSC-V2, HD, and CMK show that the proposed method achieves competitive recognition with compact learnable parameter counts of 64.05 K and 68.14 K in the 12-class and 20-class settings, respectively. The results further show that the delay-aware classifier enhances robustness under degraded acoustic conditions and effectively integrates temporally distributed evidence in compact spiking models. Together with the FPGA-oriented analytical discussion, these findings suggest that the proposed CSNN with learnable synaptic delays offers a balance between recognition accuracy and model size for edge-oriented keyword spotting.

Several limitations should also be noted. First, the hardware-oriented analysis remains a model-level estimate rather than a complete FPGA implementation. Although the sparse-computation discussion is supported by spike activity ratios measured from software inference on the test sets, these ratios cannot substitute for board-level measurements of latency, throughput, resource utilization, power consumption, and energy per inference. Thus, the current evidence supports the compactness and analytical efficiency potential of the proposed CSNN, while actual edge-device efficiency remains to be validated through hardware implementation. Second, the robustness evaluation focuses on SNR-controlled additive noise, whereas practical always-on KWS scenarios may also involve reverberation, far-field recording, and non-stationary background noise. Third, the current ablation analysis mainly focuses on the DAFC classifier and does not yet provide a finer-grained decomposition of the encoder, backbone, delay range, kernel size, or the trade-off between model size and recognition accuracy. Future work will therefore focus on FPGA-based implementation, quantization-aware deployment, robustness evaluation under realistic acoustic conditions, and more detailed component-level ablation studies for always-on keyword spotting scenarios.

## Figures and Tables

**Table 1:** List of parameters used in the preprocess module.

Datasets	Audio Length	Window Length	Hop Size	Window Function	Mel Bins
GSC-V1 and GSC-V2	16,000	1024	160	Hanning	64
HD and CMK	32,000	1024	320	Hanning	64

**Table 2:** Layer configuration and tensor shapes of the proposed lightweight CSNN under the 20-class setting.

Layer	Type	Shape	Learnable Parameters
Input	-	[batch,101,64,1]	0
BatchNorm	Normalization	[T,batch,101,64,1]	0
2D-Conv_L1	Convolution	[T,batch,97,60,8]	208
Neuron_1	LIF	[T,batch,97,60,8]	0
MaxPool_L1	Max Pooling	[T,batch,48,30,8]	0
2D-Conv_L2	Convolution	[T,batch,44,26,32]	6432
Neuron_2	LIF	[T,batch,44,26,32]	0
MaxPool_L2	Max Pooling	[T,batch,22,13,32]	0
2D-Conv_L3	Convolution	[T,batch,18,9,64]	51,264
Neuron_3	LIF	[T,batch,18,9,64]	0
MaxPool_L3	Max Pooling	[T,batch,9,4,64]	0
Reshape	Dimension Transform	[T,batch,1,256,9]	0
Padding	Padding	[T,batch,1,256,17]	0
1D-DCLS	1D-Dilated Convolution	[T,batch,1,20,13]	10,240
Temporal Aggregation	Summation	[T,batch,1,20]	0
Neuron_L4	LIF	[T,batch,1,20]	0
Readout	Spike Count	[batch,1,20]	0

**Table 3:** Classifier variants used in the CMK classifier-level ablation.

Classifier	Operation for the 64×9×4 Input	Trainable Parameters in the Classifier	Classifier Description
CFC	Flatten 2304→20 classifier	2304×20=46.10 K	Conventional fully connected classifier
Temporal convolution	2D convolution, kernel 20×3×3→ Global Average Pool	64×20×3×3=11.54 K	Temporal convolution classifier
DAFC with fixed random delays	Reshape to 256×9, pad to 256×17, one-dimensional dilated convolution	20×256=5.12 K	Delay positions are randomly initialized and fixed, synaptic weights are trained.
DAFC with learnable delays	Reshape to 256×9, pad to 256×17, one-dimensional dilated convolution with learnable delays	(20×256)×2=10.24 K	Synaptic weights and delay positions are jointly trained.

**Table 4:** Comparison of representative SNN-based keyword spotting methods under their reported experimental settings. “Official” denotes the official split setting reported for the corresponding dataset. “Random 70/10/20” denotes an utterance-level random split with 70%, 10%, and 20% samples for training, validation, and testing, respectively. “Speaker-ind. 70/10/20” denotes a speaker-independent split in which test speakers are excluded from the training and validation sets.

Method	Dataset/ Classes	Split Setting	Input	Learnable Params (K)	Test Acc. (%)
**GSC-V1, 12-class setting**					
NLIF full SNN [22]	GSC-V1/12	Official	Log-Mel feature	220	87.9
E2E SNN [18]	GSC-V1/12	Official	1D frontend	86.5	92.2
SNN-KWS [12]	GSC-V1/12	Official	Log-Mel feature	70.1	93.0
Streamlined SNN-KWS [35]	GSC-V1/12	Official	MFCC feature	50.2	93.5
Proposed CSNN with learnable delays	GSC-V1/12	Official	Log-Mel/spike encoder	64.05	92.36±0.48
Proposed CSNN with learnable delays	GSC-V1/12	Random 70/10/20	Log-Mel/spike encoder	64.05	94.37±0.55
**GSC-V2, 12-class setting**					
ST-Attention-SNN [36]	GSC-V2/12	Random 80/20	Spike events	2170	95.1
SLAYER-RF-CNN [37]	GSC-V2/12	Not specified	Spike events	280	91.4
SpikeGRU [38]	GSC-V2/12	Official	Spike events	111	94.9
SNN-KWS [12]	GSC-V2/12	Official	Log-Mel feature	70.1	94.4
Streamlined SNN-KWS [35]	GSC-V2/12	Official	MFCC feature	50.2	95.0
Proposed CSNN with learnable delays	GSC-V2/12	Official	Log-Mel/spike encoder	64.05	92.49±0.40
Proposed CSNN with learnable delays	GSC-V2/12	Random 70/10/20	Log-Mel/spike encoder	64.05	92.87±0.29
**HD, 20-class setting**					
Cuba-LIF [39]	HD/20	Official	Spike events	140	87.80±1.10
SNN with Delay [40]	HD/20	Official	Spike events	100	90.43
Adaptive Delays [41]	HD/20	Official	Spike events	100	92.45
DCLS-Delays(2L-1KC) [19]	HD/20	Official	Spike events	200	95.07
Proposed CSNN with learnable delays	HD/20	Official	Log-Mel/spike encoder	68.14	94.92±0.65
Proposed CSNN with learnable delays	HD/20	Random 70/10/20	Log-Mel/spike encoder	68.14	99.10±0.79
**CMK, 20-class setting**					
SNN with Delay [40]	CMK/20	Random 70/10/20	Spike events	100	92.17±0.32
DCLS-Delays(2L-1KC) [19]	CMK/20	Random 70/10/20	Spike events	200	94.68±0.67
Proposed CSNN with learnable delays	CMK/20	Speaker-ind. 70/10/20	Log-Mel/spike encoder	68.14	93.45±0.50
Proposed CSNN with learnable delays	CMK/20	Random 70/10/20	Log-Mel/spike encoder	68.14	95.60±0.61

**Table 5:** Analytical storage estimates of the proposed CSNN under fixed bit-width.

Item	12-Class Setting	20-Class Setting	Remark
Parameter memory (8-bit)	64.05 KB	68.14 KB	Fixed-point storage estimate
Parameter memory (16-bit)	128.10 KB	136.28 KB	Fixed-point storage estimate
Neuron-state memory (16-bit)	182.7 KB		93,556 membrane states
Bit-packed spike buffers	about 14.2 KB		Major intermediate spike maps
Full log-Mel buffer (16-bit)	about 12.6 KB		Upper-bound estimate for 101×64 features
Practical on-chip working set	about 270–300 KB		Model-level estimate with streamed execution

**Table 6:** Measured spike activity ratios of the main spike tensors on four datasets. The activity ratios were measured on the corresponding test sets using the best validation checkpoints. $\rho_{1}$, $\rho_{2}$, and $\rho_{3}$ denote the average spike activity ratios of the Conv_L2 input spike tensor, Conv_L3 input spike tensor, and DAFC input spike tensor, respectively.

Dataset	Conv_L2 Input $\rho_{1}$ (%)	Conv_L3 Input $\rho_{2}$ (%)	DAFC Input $\rho_{3}$ (%)
GSC-V1	24.84	30.60	19.64
GSC-V2	26.83	28.42	20.23
HD	16.48	15.00	11.55
CMK	23.93	20.91	10.71

## Data Availability

The public datasets used in this study are available from their original sources. The self-collected CMK dataset and the materials needed to reconstruct the reported splits are available from the corresponding author upon reasonable request, subject to institutional approval and participant privacy constraints.
